# The mediating effect of dyadic coping between illness perception and fear of disease progression in bladder cancer patients: A cross-sectional survey

**DOI:** 10.1097/MD.0000000000044520

**Published:** 2025-09-12

**Authors:** Yousheng Liu, Jingyi Li, Guanmian Liang, Fang fang, Song Wang

**Affiliations:** a Urology Department, Zhejiang Cancer Hospital, Hangzhou, China; b Nursing Department, Zhejiang Cancer Hospital, Hangzhou, China.

**Keywords:** bladder cancer, dyadic coping, fear of disease progression, illness perception, mediating effect

## Abstract

This study examines determinants of fear of disease progression (FoP) in bladder cancer patients and test dyadic coping (DC) as a mediator between illness perception (IP) and FoP. In this cross-sectional study, 126 bladder cancer patients underwent comprehensive assessment using a general information questionnaire, a fear of disease progression questionnaire-short form (FoP-Q-SF), a brief IP questionnaire and a DC inventory. Structural equation modeling with bootstrap resampling (n = 5000 iterations) quantified DC’s mediating role in the IP-FoP pathway. The scores for IP, DC, and FoP in patients with bladder cancer were (37.73 ± 12.28), (125.18 ± 23.51), and (31.18 ± 9.46), respectively. Differences in the level of fear of disease progression across gender, age, literacy, income level, time of diagnosis, whether or not they relapsed, and the number of relapses were statistically significant (all *P *< .05). Mediation analysis demonstrated that DC functioned as a significant partial mediator in the IP-FoP relationship (indirect effect *β* = 0.328, 95% CI [0.227–0.452]), accounting for 59.6% of the total effect. IP exerts both direct effects on FoP and indirect effects mediated through DC. Consequently, clinicians should implement evidence-based dyadic interventions to mitigate cancer recurrence anxiety and enhance quality of life.

## 1. Introduction

Bladder cancer represents a prevalent malignancy of the urinary system, accounting for approximately 3% of all new cancer cases globally.^[1]^ In China, 92,900 incident cases were reported in 2022, ranking it the 8th most common malignancy among males.^[2]^ non-muscle invasive bladder cancer constitutes approximately 75% of initial diagnoses.^[3]^ Primary management involves Transurethral Resection of Bladder Tumor (TURBT) with adjuvant intravesical chemotherapy. Despite favorable overall survival, non-muscle invasive bladder cancer exhibits high recurrence rates (31%–78% within 5 years).^[4]^ Repeated TURBT procedures frequently induce psychological sequelae including anxiety, fear, and helplessness.^[5]^ The 5-year survival rate for metastatic bladder cancer has declined to approximately 5%, with recurrence and metastasis being primary determinants of mortality.^[6]^

Fear of disease progression (FoP), characterized by apprehension regarding disease recurrence or advancement, is prospectively documented to be more severe in bladder cancer patients than in those with other urological malignancies.^[7]^ Illness perception (IP) constitutes a significant determinant of FoP.^[8]^ Dyadic coping (DC) encompasses the interdependent strategies through which patients and spouses collectively manage illness-related stressors.^[9]^ Empirical evidence indicates that positive DC mitigates disease-related distress and depression in oncology patients, whereas negative DC adversely affects relational intimacy and quality of life.^[10]^

This study demonstrates that FoP is modulated by multiple factors, with DC functioning as a partial mediator of the relationship between IP and FoP. Specifically, IP not only directly influences FoP but also indirectly affects its severity via DC mechanisms.

## 2. Objects and methods

### 2.1. Objects

A consecutive sample of bladder cancer patients was recruited from the Department of Urology at Zhejiang Cancer Hospital between November 2024 and April 2025. Inclusion criteria comprised: histopathologically confirmed bladder cancer; cohabiting spouse serving as primary caregiver (≥12 hours/day); cognitive competence and communication capacity sufficient for study participation; voluntary participation in the study and signing an informed consent form. Exclusion criteria: patients or spouses had a history of mental illness; nondisclosure of diagnosis to the patient.

Based on structural equation modeling requirements (5–10 observations per model parameter) and accounting for a 10% attrition rate, the target sample size ranged from 121 to 242 participants. Final enrollment yielded 126 eligible patient-spouse dyads.

### 2.2. Measurement

#### 2.2.1. General information questionnaire

Designed by the researcher, including information on age, sex, education level, time of diagnosis, marital status, family history, work status, and recurrence.

#### 2.2.2. Fear of disease progression questionnaire-short form

FoP was evaluated using the validated Chinese version of the fear of disease progression questionnaire-short form (FoP-Q-SF).^[11]^ This 12-item instrument measures disease progression concerns across 2 subdomains: physical health and social/family functioning. Responses were recorded on a 5-point Likert scale ranging from 0 (“never”) to 4 (“always”), yielding a total score between 12 and 60. Higher scores indicate greater FoP severity, with scores ≥ 34 representing clinically significant FoP according to established cutoffs. The instrument demonstrated excellent internal consistency (Cronbach α = 0.883) in this cohort.

#### 2.2.3. Brief illness perception questionnaire

IP was evaluated using the Brief IP questionnaire (BIPQ) developed by Broadbent et al.^[12]^ This validated instrument comprises 9 items assessing cognitive and emotional representations of illness across key domains. Participants rated 8 core items on a 0 to 10 Likert scale, with higher aggregate scores (range: 0–80) indicating stronger illness threat perception. The ninth open-response item identifying perceived illness causes was excluded from scoring. The instrument demonstrated acceptable internal consistency (Cronbach α = 0.708) in this cohort.

#### 2.2.4. Dyadic coping inventory

DC was examined by the DC inventory (DCI). It contains 2 dimensions: positive DC (stress communication, supportive DC, delegated DC and common DC) and negative DC. The Likert 5-point scale was used, with scores ranging from 1 to 5, from “rarely” to “very frequently,” with higher scores indicating higher DC ability. Cronbach’α for the scale was 0.971.

### 2.3. Data collection methods

The questionnaire survey in this study was implemented in a one-to-one format, first explaining to patients the content of the questionnaire, the significance of this survey, the purpose and other related information, unified guide language to the interpretation, for those who cannot fill in by themselves due to visual acuity, low literacy level, etc. The questionnaire needs to be read one by one to assist the patient in completing the questionnaire to avoid the use of suggestive language, and all the questionnaires were recovered on the spot.

### 2.4. Statistical methods

The data were analyzed using SPSS 29.0 software (Chicago). Continuous data were presented as mean ± standard deviation, and categorical data were shown as frequency and percentage. The t-test was used for comparison between groups; the correlation between disease perception, DC, and FOP was analyzed by Pearson correlation analysis; the influencing factors were analyzed by multivariate linear regression; and the mediation effect test was performed using the Bootstrap method. A 95% confidence interval (CI) not containing 0 was considered to indicate the presence of a mediating effect. *P *< .05 indicates statistical significance.

## 3. Results

### 3.1. DCI, BIPQ and FoP-Q-SF scores of bladder cancer patients

A total of 126 patients with bladder cancer were included in this study, with a DCI score (125.18 ± 23.51), BIPQ (37.74 ± 12.28) and FoP-Q-SF (31.18 ± 9.46) (Table 1).

**Table 1:** DCI, BIPQ, and FoP-Q-SF scores of bladder cancer patients (n = 126).

Subjects	Scores	Average score
Total DCI score	125.18 ± 23.51	3.58 ± 0.67
Stress communication	27.94 ± 4.60	3.49 ± 0.58
Supportive coping	31.13 ± 7.24	3.11 ± 0.72
Authorizing coping	25.82 ± 18.98	6.46 ± 4.75
Negative coping	31.48 ± 4.89	3.94 ± 0.61
Shared coping	16.32 ± 4.09	3.26 ± 0.82
Total BIPQ score	37.74 ± 12.28	4.72 ± 1.54
Cognitive	20.98 ± 8.88	4.20 ± 1.78
Emotions	12.25 ± 5.38	6.13 ± 2.69
Comprehension	4.52 ± 2.74	4.52 ± 2.74
FoP-Q-SF total score	31.18 ± 9.46	2.60 ± 0.79
Physical health	17.57 ± 4.77	2.93 ± 0.80
Social family	13.61 ± 6.13	2.27 ± 1.02

BIPQ = brief illness perception questionnaire, DCI = dyadic coping inventory, FoP-Q-SF = fear of progression questionnaire-short form.

### 3.2. Univariate analysis of general information and FoP-Q-SF scores

The results of unifactor analysis showed that the differences in scores among

Sex, age, education level, income level, diagnosis time, recurrence, and number of recurrences were statistically significant (all *P* < .05), as shown in Table 2.

**Table 2:** Univariate analysis of bladder cancer patients’ FoP (n = 126).

Project	Number of cases	FoP	*t*/*F*	*P*
Sex				
Male	105	32.12 ± 9.72	2.552	.025
Female	21	26.48 ± 6.28		
Age				
<60 yr old	40	37.43 ± 8.74	23.949	<.001
60–80 yr old	72	29.67 ± 8.25		
>80 yr old	14	21.14 ± 4.28		
Educational level				
Junior high school and below	108	29.66 ± 8.90	12.225	<.001
High school or junior college	13	41.77 ± 7.79		
College and above	5	36.60 ± 5.81		
Per capita monthly household income (yuan)				
<3000	30	36.90 ± 7.24	10.607	<.001
3000–6000	65	30.52 ± 9.51		
>6000	31	26.70 ± 8.66		
Work situation				
Working	41	36.68 ± 9.00	4.939	.661
nonworking	85	28.53 ± 8.53		
Type of medical insurance				
Employee medical insurance or public funds	31	32.58	0.480	.620
Agricultural insurance	91	30.78		
Self-funded	4	29.50		
Place of residence				
Rural	99	31.20 ± 9.91	0.044	.104
Urban	27	31.11 ± 7.73		
Number of children				
1	1	32.00	0.161	.851
1–2	114	31.32 ± 9.66		
≥3	11	29.64 ± 7.87		
Family tumor history				
No	93	31.54 ± 9.50	0.706	.808
Yes	33	30.18 ± 9.43		
Time to diagnosis				
<3 mo	75	27.76 ± 7.40	22.379	<.001
3 mo–3 yr	35	33.51 ± 9.38		
>3 yr	16	42.13 ± 8.79		
Recurrence				
yes	43	38.81 ± 9.19	7.989	.035
No	83	27.23 ± 6.84		
Number of relapses				
0 times	86	27.28 ± 6.73	34.919	<.001
1 time	11	32.73 ± 9.48		
2–4 times	22	40.27 ± 6.68		
≥5 times	7	48.14 ± 7.47		

FoP = fear of disease progression.

### 3.3. Correlation between dyadic coping, disease perception and fear of disease progression in bladder cancer patients

DC ability was negatively correlated with disease perception and recurrence fear level (*r* = −0.663, −0.833, both *P* < .001), and disease perception was positively correlated with recurrence fear level (*R* = 0.714, *P* < .001) (Table 3).

**Table 3:** Correlation analysis of DCI, BIPQ and FoP-Q-SF in bladder cancer patients.

Projects	DCI	Stress communication	Support response	Authorized response	Negative coping	Shared coping	BIPQ	Cognitive	Emotions	Comprehension	FoP-Q-SF	Physical	Social family
DCI	1	–	–	–	–	–	–	–	–	–	–	–	–
Stress communication	0.462*	1	–	–	–	–	–	–	–	–	–	–	–
Support response	0.467*	0.663*	1	–	–	–	–	–	–	–	–	–	–
Authorized response	0.639*	0.391*	0.016	1	–	–	–	–	–	–	–	–	–
Negative coping	−0.376*	−0.253*	−0.124	−0.552*	1	–	–	–	–	–	–	–	–
Shared coping	0.582*	0.764*	0.669*	0.418*	−0.385*	1	–	–	–	–	–	–	–
BIPQ	−0.663*	−0.490*	−0.263*	−0.753*	0.532*	−0.566*	1	–	–	–	–	–	–
Cognitive	−0.598*	−0.382*	−0.242*	−0.581*	0.387*	−0.426*	0.877*	1	–	–	–	–	–
Emotions	−0.301*	−0.327*	−0.052	−0.520*	0.333*	−0.355*	0.596*	0.197**	1	–	–	–	–
Comprehension	−0.443*	−0.313*	−0.294*	−0.469*	0.478*	−0.456*	0.470*	0.300*	0.067	1	–	–	–
FoP-Q-SF	−0.833*	−0.361*	−0.319*	−0.600*	0.354*	−0.524*	0.714*	0.654*	0.373*	0.348*	1	–	–
Physical health	−0.704*	−0.299*	−0.255*	−0.458*	0.291*	−0.467*	0.580*	0.562*	0.234*	0.319*	0.827*	1	–
Social family	−0.737*	−0.324*	−0.294*	−0.569*	0.320*	−0.445*	0.649*	0.571*	0.393*	0.288*	0.899*	0.498*	1

BIPQ = brief illness perception questionnaire, DCI = dyadic coping inventory, FoP-Q-SF = fear of progression questionnaire-short form.

* *P*<.01.

** *P*<.05.

### 3.4. Mediating effect of dyadic coping between disease perception and fear of disease progression in bladder cancer patients

**Figure 1.**
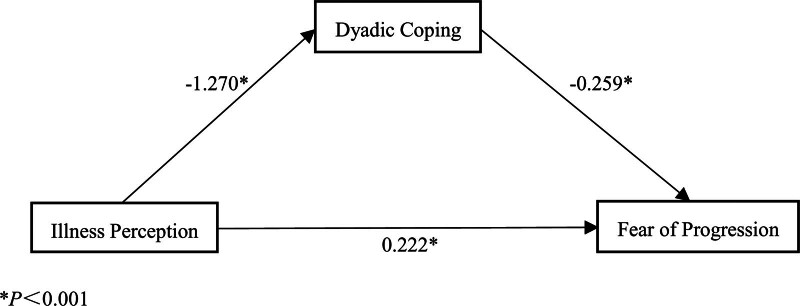
Dyadic coping in bladder cancer patients mediates pathways between disease perception and FoP. *P<.001. FoP = fear of disease progression.

By using Model4 in the SPSS (Chicago) macro program process to test the mediating effect, with BIPQ as the independent variable, FoP as the dependent variable, and DCI as the mediating variable, 3 regression path models were obtained according to bootstrap method analysis. As shown in Table 4, the value of the direct effect is 0.222, with an effect share of 40.36%; the value of the indirect effect is 0.328, with an effect share of 59.64%; and the CIs of the direct and indirect effects do not contain 0, indicating that the effects are valid. The path coefficients of the variables are presented in Figure 1. Thus, disease perception can directly affect the level of fear of disease progression, and it can also indirectly affect the level of fear of disease progression through DC skills.

**Table 4:** Mediation effect test of DCI between BIPQ and FoP.

Project	Effect	*SE*	95% CI	*P*	Relative effect (%)
Total effect	0.550	0.049	0.454–0.646	<.001	–
Direct effect (BIPQ→FoP)	0.222	0.047	0.128–0.315	<.001	40.36
Indirect effect (BIPQ→DCI→FoP)	0.328	0.060	0.227–0.452	<.001	59.64

BIPQ = brief illness perception questionnaire, CI = confidence interval, DCI = dyadic coping inventory, FoP = fear of disease progression, SD = standard deviation.

## 4. Discussion

### 4.1. Analysis of the situation of bladder cancer patients’ FoP and influence factors

The results showed that the FoP-Q-SF score of bladder cancer patients was (31.18 ± 9.46), which was at a high level, similar to the results of the study of bladder cancer patients by Xiao et al,^[13]^ and higher than that of the cross-sectional investigation of patients with chronic heart failure by Xiong et al^[14]^ and that the high recurrence and metastatic characteristics may be an important factor that leads to a high level of FoP. Even after completion of TURBT surgery and postoperative bladder perfusion therapy, patients still need to face long-term recurrence monitoring (e.g., cystoscopy every 3–6 months), and this “treatment-recurrence-retreatment” cyclic pattern puts patients in a long-term perception of the threat of disease progression. In addition, 38.1% of the patients in this study had psychological dysfunction (FoP-Q-SF ≥ 34 points), which was higher than that in Stewart’s^[15]^ study of patients with cancer recurrence. The results of this study showed that gender, age, education, income level, time of diagnosis, recurrence, and the number of recurrences were the influencing factors of FoP. Possible reasons: influenced by traditional gender roles, men tend to repress emotions and avoid preventive medical care, whereas women are more likely to seek social support. This gender difference may lead to men accumulating anxiety and a sense of uncontrollability regarding recurrence over time. Young patients have higher expectations of their future health and are in a critical period of career development and family responsibilities. Disease progression increases the difference in life expectancy and disrupts patients’ long-term careers and family planning. In addition, young patients are more concerned about the impact of surgery on fertility and physical intentions, which can easily cause anxiety and fear. Highly educated people are more sensitive to information, interpret symptoms on their own based on fragmented medical knowledge, amplify sensitivity to minor abnormalities, and overly focus on recurrence rate and poor prognosis, leading to an amplification of recurrence risk. Low-income families may have fewer social resources and lack social support networks. Multiple surgeries and regular bladder perfusion chemotherapy can directly increase the financial burden, whereas high-income families are more risk-resistant and usually have commercial insurance supplements, leading to lower FoP scores. Patients who have been diagnosed for a long time may experience multiple relapses and fluctuations and are in a long-term state of alertness to the “threat of the disease.” Multiple relapses gradually change the patient’s goal of treatment from curing to minimizing relapses, which reinforces the patient’s pessimistic expectations, and in addition, the long-term illness leads to the shrinkage of the social circle, which is prone to the stripping away of the social roles.

### 4.2. FoP in bladder cancer patients is strongly associated with IP and DC

In this study, the DCI score of bladder cancer patients was (125.18 ± 23.51), which was at an intermediate level and higher than that reported by Cai et al^[16]^ on breast cancer partners. This discrepancy may reflect older demographic profiles facilitating established relational stability, longer cohabitation duration enabling dyadic schema development, and disease recurrence patterns necessitating adaptive coping mechanisms. The results of the correlation analysis showed that FoP was negatively correlated with DC (*r* = −0.883, *P* < .001), indicating that the higher the DC ability between partners, the lower the level of fear of disease progression. Among them, negative DC positively predicted the level of fear of disease progression, and stress communication, supportive coping, empowering coping, and coping together negatively predicted fear of disease progression, which is similar to the findings of Ma.^[17]^ This also supports the idea of the intimacy model that interaction is the basis of intimacy, that self-representation and partner response are key components of intimate interaction, and that perceived partner response mediates the role of self-representation and intimacy, which can be effective in mitigating the negative stimulation of individuals by disease stressors.^[18]^ Therefore, healthcare professionals need to go beyond the traditional “patient-centered” model, consider both partners to reduce the level of fear of disease progression.

In this study, the BIPQ score was (37.74 ± 12.28), which was at a high level, probably related to the high recurrence rate of bladder cancer and the lower cognitive level caused by the patients’ higher average age and limited literacy level; the total IP score and scores of the dimensions were positively correlated with the FoP (*R* = 0.714, *P* < .001), indicating that the greater the perceived threat of the disease, the more fearful the disease progression is, which is consistent with the findings of Wang Xiang^[19]^ and Wang X et al.^[20]^ Therefore, healthcare professionals can use short videos and visualization tools for disease education to reshape the cognitive framework of disease perception on the 1 hand, and on the other hand, they can provide emotional support through peer support, DC, and nurse-patient communication to enhance patients’ sense of disease control. In addition, the results of this study also showed that DC was negatively correlated with IP (*r* = −0.663, *P* < .001); patients perceiving the threat of the disease may repeatedly ask for evidence due to the fear of recurrence, the partner chooses to emotionally avoid it due to the feeling of powerlessness, or the patient conceals the symptoms due to the unwillingness to aggravate the burden of the partner, and the continuous coping with the high threat of the disease is likely to lead to adopting ineffective coping. Poor communication often increases negative perceptions of illness.

### 4.3. DC partially mediates between IP and FoP

This study establishes DC as a significant partial mediator between IP and FoP in bladder cancer patients (indirect effect *β* = 0.328, 95% *CI* [0.227–0.452]). DC accounted for 59.64% of the total effect, indicating that IP influences FoP both directly and indirectly through dyadic pathways.

IP constitutes a primary determinant of FoP severity, wherein threatening illness representations correlate positively with heightened FoP levels.^[21]^ Empirical evidence confirms IP’s robust influence on DC dynamics.^[22,23]^ According to the Systemic Transactional Model,^[24]^ partners communicate perceived stress through verbal/nonverbal channels, triggering reciprocal cognitive appraisal and subsequent engagement in either adaptive or maladaptive DC. These dyadic processes directly modulate both partners’ psychological well-being. Critically, positive DC mitigates psychiatric morbidity and enhances quality of life.^[25]^ This suggests that healthcare professionals should pay attention to the mediating effect of DC, while improving the perception of illness in bladder cancer patients. They should also establish a family healthcare collaborative network and adopt personalized interventions such as narrative therapy, duo-care skills workshops, and division of labor task management to improve DC, in order to reduce the level of fear of disease progression.

## 5. Conclusion

Bladder cancer patients exhibit clinically significant FoP, with IP demonstrating both direct effects on FoP and indirect effects mediated through DC, suggesting that healthcare professionals can formulate a coping strategy based on the DC perspective. This study has several constraints, including single-center recruitment potentially limiting generalizability and a large difference in the proportion of male and female sample sizes, which has a certain impact on the results of the study, so multi-center and large-sample studies can be carried out in the future; in addition, this study is a cross-sectional investigation, future work should employ longitudinal cohorts to establish temporal dynamics. The findings of this study are particularly applicable to healthcare organizations with similar diagnostic and treatment conditions. Further validation is needed for areas with large differences in healthcare resources before replication.

## Author contributions

**Conceptualization:** You Sheng Liu.

**Data curation:** Jingyi Li.

**Formal analysis:** Jingyi Li.

**Investigation:** Jingyi Li, Fang Fang.

**Methodology:** You Sheng Liu, Jingyi Li, Song Wang.

**Project administration:** You Sheng Liu, Guanmian Liang.

**Supervision:** You Sheng Liu, Guanmian Liang.

**Validation:** Jingyi Li.

**Visualization:** Guanmian Liang.

**Writing – original draft:** You Sheng Liu, Jingyi Li.

**Writing – review & editing:** You Sheng Liu, Jingyi Li, Song Wang.
